# Comparison of Adherence to Mediterranean Diet between Spanish and German School-Children and Influence of Gender, Overweight, and Physical Activity

**DOI:** 10.3390/nu14214697

**Published:** 2022-11-07

**Authors:** Lena Grams, Anne-Katrin Nelius, Guadalupe Garrido Pastor, Manuel Sillero-Quintana, Óscar L. Veiga, Denise Homeyer, Momme Kück

**Affiliations:** 1 Faculty of Science, Physical Activity and Sports (INEF), Technical University of Madrid (UPM), 28040 Madrid, Spain; 2 Institute of Sports Medicine, Hannover Medical School, 30625 Hannover, Germany; 3 Department of Physical Education, Sport and Human Movement, Autonomous University of Madrid (UAM), 28049 Madrid, Spain

**Keywords:** nutrition, mediterranean diet, kidmed, physical activity, adolescents, body composition

## Abstract

Background: Poor dietary habits and low levels of physical activity (PA) have a strong tendency to track from childhood into adulthood. The Mediterranean Diet (MD) is known to be extremely healthy, associated with lower BMI and a lower risk of obesity in children and adolescents. Therefore, adherence to the MD was compared between Spanish (*n* = 182) and German (*n* = 152) children aged 10 to 13 years to examine a possible more “westernized” diet in Spain with a non-Mediterranean country, that traditionally prefers a “Western diet” and to determine the association between adherence to the MD and gender, body composition, and PA levels. Methods: In the German observational longitudinal cohort study and the Spanish cohort study, body composition and questionnaires (KIDMED, Diet Quality (IAES)) were obtained, and accelerometers (Actigraph) were applied to detect PA. Results: Girls had higher BMI-standard deviation score (SDS) than boys and Spanish girls were less active than boys. Differences were detected in MD habits, such as favorable fruit-, vegetables-, fish-intakes, and dairy products in Spanish children and unfavorable consumptions of fast food, processed bakery goods, candies, and sweet beverages in German children. Independently of country, girls, children with lower BMI-SDS and children with higher PA level were related with better diet quality. Conclusion: Spanish children showed higher adherence to MD and diet quality (IAES) compared to German children, but there was a trend toward a more “westernized” diet. Gender, body composition, and PA influenced nutrition regardless of country.

## 1. Introduction

Overweight and obesity are a global burden and a tremendous problem for children and adolescents, with one in three children living with overweight or obesity [1]. Being overweight or obese seems to be the result of several factors and the causes are still not known entirely [2]. However, one reason is an unhealthy lifestyle behavior, such as poor dietary habits, high sedentary time, and low levels of physical activity (PA), which has a strong tendency to track from childhood into adulthood [3,4]. Bull et al. concluded that PA has benefits for various health outcomes, such as physical fitness, cardio-metabolic health, bone health, cognitive outcomes, mental health, and reduced adiposity [5]. To achieve this, the World Health Organization (WHO) recommends for children and adolescents (aged 5–17 years) at least an average of 60 min/day of moderate to vigorous intensity PA. Amounts of sedentary time is recommended to be reduced, especially screen time.

In addition to PA, nutrition plays a key role in health and is closely related. A healthy diet is defined by the WHO as health-promoting and disease-preventing [6]. It provides adequacy without excess of macro- and micro-nutrients from nutritious foods and avoids the consumption of health-harming substances. A nutritious food is described as “one that provides beneficial nutrients (e.g., protein, vitamins, minerals, essential amino acids, essential fatty acids, or dietary fiber) and minimizes potentially harmful elements (e.g., quantities of sodium, saturated fats, sugars)” [7]. The combination of different foods in a diet and the quantity, variety, and frequency with what they are consumed are defined as dietary pattern. These patterns may be traditional or based on a special interest, such as a plant-based diet without animal products.

The “Mediterranean diet” (MD) is traditionally the preferred dietary pattern in Spain. It is high in plant-based foods, such as fresh fruits and vegetables, whole grains, legumes, seeds, and nuts and low in animal-based foods, mainly fatty and processed meats [8]. Olive oil is its principal source of added fat and high to moderate intakes of fish and seafood. Eggs and white meat should be eaten moderately (2–4 times per week) [9]. The MD is characterized by a low content of saturated fatty acids and high in mono-saturated fatty acids, together with high amounts of fiber and complex carbohydrates. Additionally, several social and gastronomical aspects characterize a particular lifestyle. The MD combines ingredients from local agriculture, recipes, and traditional cooking methods [10], which are also the same principles of sustainable nutrition. Furthermore, a low red meat intake should be beneficial to maintain a healthy planet nowadays, as meat production accounts for the most significant adverse environmental impact on Earth [11].

Even though MD is the traditional diet pattern, the adherence to it is only moderate in the Mediterranean adult population in the past 10 years [12] and the same trend toward a more “westernized” diet can be observed among children and adolescents in Mediterranean countries [13]. In contrast to MD, the “Western diet” is rather low in fruits and vegetables, and high in fat and sodium. It consists of large portions, with high amounts of energy, excess sugar, low fiber, and high saturated and trans fats, which increase calories and low-density lipoproteins, resulting in an increased risk of developing atherosclerosis [14].

Consequences of low levels of PA and poor nutrition in adolescents have become important topics of scientific interest in Spain and Europe. Results from the Spanish HELENA (Healthy Lifestyle in Europe by Nutrition in Adolescence) study proved that being physically active is the most significant and protective outcome in adolescents to reduce cardio-metabolic risk [15]. Results from another Spanish study found that 25% are overweight and 10% suffer from obesity in children aged 8–13 in 2012 [16]. In Germany, where the “Western diet” is more common, data from the KIGGS (German Health Interview and Examination Survey for Children and Adolescents) study in Germany demonstrate that 15% of children and adolescents aged 3–17 years are overweight and 6% suffer from obesity [17]. Börnhorst et al. found that one-third of the children between 2 and 9 years old, who participated in the European study IDEFICS (Identification and prevention of Dietary- and lifestyle-induced health EFfects In Children and infantS), did not reach the guidelines (D-A-CH Reference Values for the European Population) for carbohydrate intake (50%) and exceeded the recommendations for fat intake (30–35%) [18]. Less than 10% reached the recommendations for water intake of 1 L per day. Except for Estonia, sugar intake in all other participating countries (Germany, Belgium, Italy, Sweden, Spain, Hungary, and Cyprus) exceeded the recommendations by 20%, and in Germany, by 30% [19].

Previous research indicates that the adherence to the principles of MD is associated with lower BMI and a lower risk of obesity in children and adolescents [20,21], and that its beneficial implications both for clinical and environmental aspects should be exported to other countries [22]. Therefore, the adherence to MD was compared between Spanish and German children aged 10 to 13 years to examine a possible more “westernized” diet in Spain with a non-Mediterranean country, that traditionally prefers a “Western diet”, and to determine the association between adherence to MD and gender, body composition, and PA levels.

## 2. Methods

Baseline data from two separate studies in Spain and Germany were analyzed. In the Spanish cohort study, 182 children participated from six public schools in Madrid’s central area, with high sociocultural status and ethnicity variability. The district and schools were selected by the funder, since the foundation (Fundación Maratón) was looking for schools to promote physical activity and health in critical environment. To collect data on nutrition and physical activity, they contacted the Technical and Autonomous University of Madrid for health professionals. Data were obtained between October and December 2015. Participating children and their parents gave written consent before initiating the study, which was approved by the ethics committee of the Universidad Politécnica de Madrid (UPM, PIA 12009-11, Date: 5 March 2015) and based on the guidelines of the Helsinki Declaration. One hundred fifty-two children from two secondary schools in northern Germany (Lower Saxony and North Rhine-Westphalia) participated in the German observational longitudinal cohort study “Rebirth active school”, which also showed high variability in sociocultural status, but took place in cities smaller than Madrid. As “Rebirth active school” was a pilot study, secondary schools were asked to participate. The overall goal was to integrate a physical activity program into the school day, achieving WHO recommendations on PA. Further details are published by Memaran et al. [23]. Data were obtained between April and June 2017 as baseline of an overall 3-year study period. Participating children and their parents gave written consent, and the study was approved by the Hannover Medical School ethics committee (Number 1790, Date: 9 January 2017), based on the guidelines of the Helsinki Declaration. Inclusion criteria in both studies was school affiliation. Exclusion criteria included being at home on the day of data collection.

### 2.1. Anthropometrics

In both studies, height and weight were measured using a portable stadiometer (Spain: SECA 213, SECA, Azcapotzalco, Mexico; SECA 764, Seca GmbH & Co. KG, Hamburg, Germany) in light clothes without shoes. Hip circumference was measured with a standard centimeter. All anthropometric measurements were recorded according to the International Society for the Advancement of Kinanthropometry principles [24]. Each measurement was carried out at least two times and, if the percentage of measurement error was insufficient (>1%), a third measurement was performed. For two measurements, the mean was taken as the outcome variable, and for three measurements, the median [24].

According to the German population, height, weight, BMI, and hip circumference were transformed to standard deviation scores (SDS) to compare the two countries normalized by age and gender [25]. “Overweight” was defined as BMI-SDS over 1.282 and “obese” as BMI-SDS over 1.881 [26]. Therefore, three groups “normal weight”, overweight”, and “obese” were used in the nutrition analysis. For PA analysis, “overweight” and “obese” were combined to one group, and two groups “normal weight” and “overweight” were used. In both studies, all measurements were performed by previously trained medical personnel or sports scientists in a one-to-one situation.

### 2.2. Questionnaires

To assess the adherence to the Mediterranean diet in children, the Mediterranean Diet Quality Index or KIDMED was used [27]. KIDMED was the first to assess adherence to Mediterranean dietary patterns in children and youth [27]. The psychometric investigating properties of this index have also been used in other countries not classified as Mediterranean geographies, such as Portugal [28]. It consists of 16 questions that are answered “yes” or “no”. Thirteen questions have a positive aspect (score +1) and three a negative aspect (score −1). The KIDMED is the sum of these scores, ranging from −3 to 13. It is categorized as “very low or deficient quality” (KIDMED ≤ 3), “need to improve the eating pattern to adjust it to the Mediterranean model” (KIDMED from 4 to 7), and “optimal Mediterranean diet” (KIDMED ≥ 8). To compare the questionnaires of both projects, three questions with a positive aspect had to be skipped (nuts intake, rice and starchy food, and the usage of olive oil at home). They have been deleted for the German study since, for example, most German children did not know, which oil was being used for cooking at home. All other questions were part of both studies. Therefore, the KIDMED ranged from −3 up to 10.

The Index of a healthy Alimentation diet for the Spanish population (IASE) is a rapid and affordable method to estimate the quality of the diet in the Spanish population [29]; it uses secondary data from the Spanish National Health and Nutrition Survey and the feeding guidelines. The IASE identifies the frequency intakes of food groups using 9 of the 12 food groups intake described in the Spanish National Health and Nutrition Survey (ENS-06). It includes 10 variables, 9 questions, and a variable “diet-variety” calculated from the answers to the 9 questions. The first five questions represent the consuming of the main groups of alimentations (grains and derivatives, fruits, vegetables, dairy products, and meat) and the remaining four represent the achievement of reference nutritional objectives (total fat, saturated fat, cholesterol, and socio), asking for cold-processed meats, sweets, and beverages. Every variable gets a score from 0 to 10 with higher scores representing better diet quality. The sum of the scores makes it possible to calculate the IASE with a maximum value of 100 and to classify the diet into three categories: “Healthy feeding” (score > 80), “need-for-change” (score between 50 and 80), and “little healthy” (score < 50) following the recommendations of the Spanish Feeding Guidelines [29,30].

Both questionnaires were translated into German from the scientifically published English version by native speakers and back-translated to avoid errors. The original Spanish version was used in the Spanish study. Questionnaires were completed at the beginning of each test day and each child was interviewed in a one-to-one situation by previously trained sports scientists.

KIDMED and IASE were used complementarily for in-depth analysis of dietary quality, since KIDMED was designed for use in children and adolescents, and IASE supplements additional information to the KIDMED results.

### 2.3. Accelerometers

The accelerometer models used in the Spanish study were the ActiGraph GT1M, GT3X, and GT3X+ (Actigraph TM, LLC, Fort Walton Beach, FL, USA) worn at the hip and the ActiGraph GT9X Link (Actigraph TM, LLC, Fort Walton Beach, FL, USA) worn at the non-dominant wrist in the German study. The GT1M device is a uniaxial accelerometer, while all other devices are triaxial accelerometers. The generations GT1M and GT3X+ showed strong agreement in children under free-living condition and thus were used in the Spanish study [31,32]. Instructions on how to wear the accelerometer were explained to participants on the day of device delivery. In addition, this information was provided to parents/guardians and school administrators in written form. The Spanish children wore the device for 7 consecutive days attached on an elastic band on their lower back, whereas the German children wore it for at least 3 consecutive weekdays and 1 weekend on their non-dominant wrist. All participants were asked to take off their accelerometer for water activities, such as swimming and taking a shower or when going to sleep. 

Data were downloaded and analyzed using Actilife software (version 6.13.4 in the German study, version 6.62 in the Spanish study, Actigraph TM, Pensacola, FL, USA). In the Spanish study, a daily recording duration of at least 10 h per day was required to be included in the analysis and for the classification of physical activity into light, moderate, and vigorous intensity, the cut-off points proposed in the HELENA study were used [33]. In the German study, the cut-off points from Chandler et al. were used, and at least 1152 min per day must have been recorded [34]. Mean time per day spent in light, moderate, and vigorous intensity were calculated. Since both studies used different devices, different wearing positions, and different minimum requirements for valid days, for each country the mean time per day in moderate and vigorous intensity spent during weekdays, weekend days, and all valid days was calculated separately. Then, the percentage of weekend days compared to weekdays was determined and the PA levels were sent as “low” for being in the first tertile of all valid days, “medium” for being in the middle tertile, and “high” for being in the third tertile.

### 2.4. Statistics

All data are given as mean ± standard deviation. Normal distribution was tested using the Kolmogorov-Smirnov test. Distribution of the data was tested with a Chi-squared test. Differences between the two groups were tested with an unpaired t-test for parametric data, respectively, and a Mann-Whitney-U test was used for non-parametric data with Hedges’ g as the effect size. The interaction of country and gender was computed with an ANOVA with eta squared η^2^ as the effect size. All post-hoc tests were corrected by Bonferroni’s method. Odd ratios were calculated with a forward stepwise binary logistic regression. A backwards multiple linear regression was used to determine the relationship between KIDMED and IAES as a dependent variable, and country, gender, BMI group, and PA level as independent variables. The significance level was set at 0.05. All calculations were carried out with SPSS (version 27, Armonk, NY, USA).

## 3. Results

A total of 334 boys and girls were included in the analysis, 152 from Germany and 182 from Spain. There were no significant differences between both countries (see Table 1). The girls showed higher weight, BMI, and waist circumference than the boys. The interaction between gender and country was significant for age.

**Figure 1:**
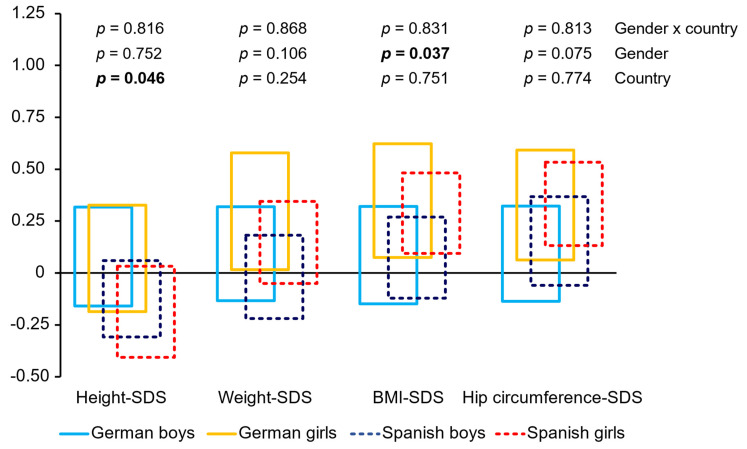
The 95% confidence intervals of SDS-scores and results of ANOVA. Bold: *p* < 0.05.

The analysis of the SDS-scores showed no significant differences for the interaction of gender and country (see Figure 1). Height-SDS differed significantly between both countries with higher values for Germany (η^2^ = 0.01) and the girls had significantly higher BMI-SDS than the boys (η^2^ = 0.01). BMI-SDS and hip circumference-SDS of German and Spanish girls were above zero.

The PA level distribution showed a significant difference for gender and BMI group in Spain. Spanish girls were less active than the boys and the overweight group was less active than the normal weight (see Table 2). The activity on weekend days was lower than on weekdays with a higher decrease in Spain than in Germany (Germany 80.4 ± 36.5%; Spain 70.3 ± 32.2%; *p* = 0.014; g = 0.30).

Table 3 shows the results of the each KIDMED question by country, with 10 out of 13 questions showing significant differences between both countries. The KIDMED score was 3.45 ± 1.99 for Germany and 6.15 ± 1.95 for Spain, with no significant differences between gender (p_ANOVA_ = 0.078) and the interaction gender and country (p_ANOVA_ = 0.486), but it differed significantly between the two countries (p_ANOVA_ < 0.001; η^2^ = 0.32).

**Figure 2:**
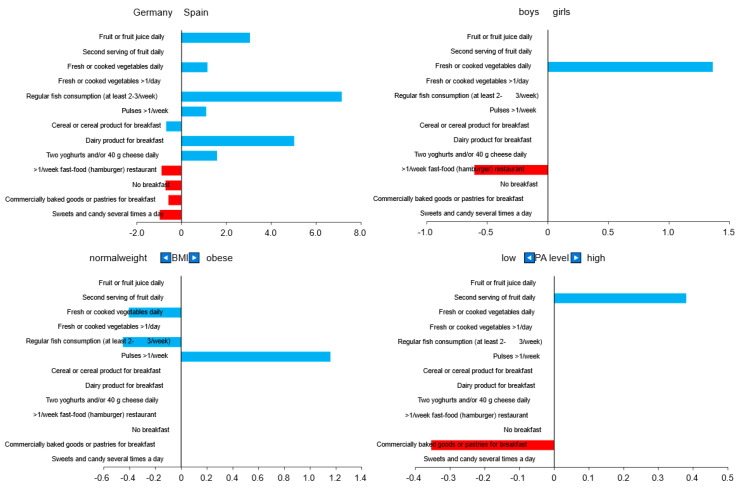
Odd ratios for each KIDMED question for country, gender, BMI group, and PA level. Blue: Questions with a positive aspect (+1); red: Questions with a negative aspect (−1). PA: Physical activity.

Odd ratios for all KIDMED questions are displayed in Figure 2. The Spanish children showed a higher probability of answering “yes” for six out of nine KIDMED questions with a positive aspect and a lower probability of answering “yes” for all questions with a negative aspect. Children with a higher PA level had simultaneously a higher probability to eat a second serving of fruits per day and a lower one to eat commercially baked goods or pastries for breakfast. The multiple linear regression showed positive relationships between Spain, girls, and higher PA level (see Figure 2).

The IASE and its variables are shown in Table 4. The IASE and nine out of ten variables were significantly higher in the Spanish children than in the German ones. The classification in the three categories was significantly different (*p <* 0.001): “Little healthy” (Germany 65.3%, Spain 0%), “need-for-change” (Germany 34.0%, Spain 52.2%), and “healthy feeding” (Germany 0.7%, Spain 47.8%).

**Figure 3:**
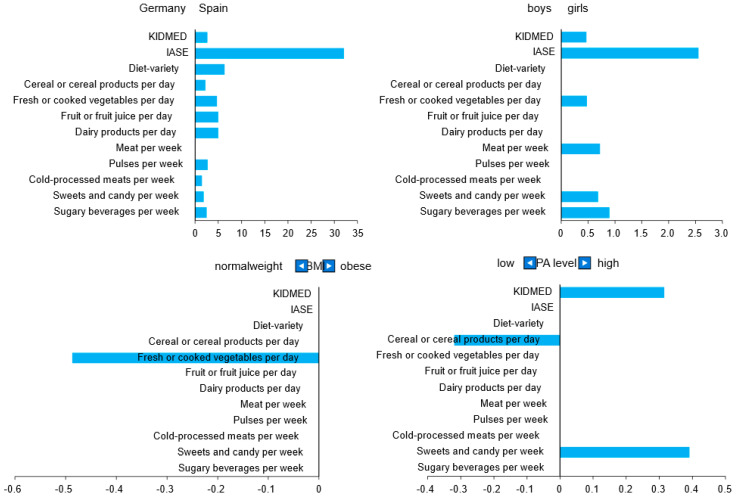
Regression coefficients for KIDMED and IASE and its variables for country, gender, BMI group, and PA level. PA: Physical activity; IASE: Healthy feeding indicator.

Figure 3 displays the results of the multiple linear regression. The IASE and nine of its variables showed a positive relationship with Spain. The girls had a positive relationship with the IASE and the BMI group had a negative relationship with fresh or cooked vegetables per day, indicating that the higher the BMI-SDS, the unhealthier the answer.

## 4. Discussion

The aim of the present study was to compare the adherence to the MD between Spain and a non-Mediterranean country in school children aged 10–13 and to determine the association between adherence to MD and gender, body composition, and PA levels. Results showed that Spanish children had a higher adherence to MD and diet quality (IAES) compared to German children, but there was a trend toward a more “westernized” diet among the Spanish children studied. Gender, body composition, and PA influenced nutrition regardless of country.

When analyzing both countries together, females reached higher results in both nutritional scores (KIDMED and IAES) compared to the males. Summarizing these results from the KIDMED and IAES, the Spanish children showed higher adherence to MD compared to German children, but although the MD is accepted and promoted in Germany, the German children reached scores well beyond their Spanish counterparts. Therefore, the probability of answering “yes” to the questions of KIDMED with a positive aspect was only higher for Spanish children and to the questions with a negative aspect only for German children. 

Similar results showed the questions for IAES, where only Spanish children had a positive relationship with healthier answers. Since three questions with a positive aspect and adherence to MD were not asked out of the original KIDMED, the results could not be categorized. Therefore, the probability of answering “yes” to the questions of KIDMED with a positive aspect was only higher for Spanish children and to the questions with a negative aspect only for German children. Similar results showed the questions for IAES, where only Spanish children had a positive relationship with healthier answers. Since three questions with a positive aspect from the original KIDMED were not asked, the results could not be categorized. However, the results showed that neither country would reach very low adherence to MD as the German children reached at least 3.45 ± 1.99. The Spanish children (6.15 ± 1.95) were closer to achieve good adherence to MD, knowing that the three missing items are typically for MD (olive oil, nuts, and grain foods). Therefore, it is rather unlikely that the German group would achieve higher scores and would “need to improve their eating pattern to adjust it to the MD” according to the reference values of KIDMED.

Data from the second wave of the German KIGGS study analyzed over 1353 adolescents, which showed inadequate consumption of fruits, vegetables, starchy, and milk/dairy products, and exceeded the intake of meat and sugar-sweetened beverages [4]. These results are in line with those of this study, compared to the Spanish children. It should be noted, however, that this study applied food-frequency questionnaires, which did not include questions regarding quantities (e.g., g/day) of food, such as in KIGGS. A review from Garcia–Cabrera et al. analyzed 18 studies (17 southern European studies and one from Chile regarding adherence to the MD in children and young adults 2–25 years) [13]. Only 10% of all 24,067 participants had a high adhesion to the MD applying the KIDMED, while 21% showed low adhesion. A study by Farajian et al., with 4786 Greek children comparable in age, showed that 45% had a low adherence to the MD and only 5% showed high adherence [35]. Lopez-Gil et al. found in 26% of his study population optimal adherence to MD, applying the KIDMED questionnaire [36]. All these results show not only the relevance of the geographical location, but also a moderate adherence to MD, which showed the need to improve the MD in these countries [37].

Similarly, the IAES data reflected the “need-for-changes” in the quality of the diet of the German children. Recommendations for a healthy diet recommend at least 5 portions of fruit and vegetable intake per day. Fruit intake was significantly higher in Spain (91% vs. 70%). Similarly, 75% of the Spanish group compared to 57% of the German group ate vegetables once per day. Children with higher PA levels consumed more often a second serving of fruits per day, regardless of country. The highest difference was displayed when analyzing the consumption of fish per week (Spain 81%, German 35%). However, in a study by Mariscal et al. [38], comparing the Spanish group to another comparable Spanish population, it appears that various intakes have been dropped (e.g., fruits, vegetables, dairy products). However, on the contrary, the consumption of fish increased from 42% up to 81%. Spain has a vast coast where fish and other sea product intakes are usual, and their availability and fair prices could be a determinant of higher consumption. It is quite common to purchase sea products from many little shops or fisheries located in the town on different areas than the supermarkets. Furthermore, in Spain, which is traditionally associated with Catholic beliefs, a meat-free diet with fish and sea products is practiced during the Fridays of Lent. Even in a town, such as Madrid, which has no coastline, the fish availability is great. Furthermore, typical Spanish recipes use sea products for cooking. These changes, some positive, but also negative, may be explained by Interventions and promotion of healthy foods in schools. Typically unhealthy intakes associated with the “Western diet” were found in German children, who had significantly higher intakes of fast food, commercially baked goods, ate sweets and candies several times per day, and skipped their breakfast more often. Biblioni et al. found the “Western” dietary pattern often among boys, while the “Mediterranean” dietary pattern was mainly followed by girls, which is in line with this study [39].

These “need for changes” for the German children are important, as meal patterns, such as skipped breakfast, have been suggested as a marker of an inappropriate dietary intake among adolescents [40]. Similarly, eating frequency was identified as a risk factor for obesity in both boys and girls [41]. Hoyland et al. found that breakfast has beneficial effects in school children on cognitive function compared to those who attend school fasting [42]. Forkert et al. found an increased waist circumference in European girls with low physical activity levels, which is an indicator of being overweight or obese [43]. When skipping breakfast, they found a strong relation between total and abdominal obesity. Poor adherence to MD was shown to be closely related to obesity in Greek school-children [44]. Flieh et al. found a strong relationship between free sugar intake and obesity in 843 European adolescents [45].

In Spain, it has been described that more than 80% of all ultra-processed food add sugar [46]. Specifically, children consuming higher amounts of sweet beverages demonstrated significantly increased amounts of LDL, tryglyceride, and cholesterol and lower amounts of HDL [47]. More specifically, Hebestreit et al. mentioned the association between daily energy intake and age-gender-specific BMI, which should be detected in future studies, since a relationship seems to be noticeable [48].

The results on obesity and overweight did not show significant differences between German and Spanish children. According to the KIGGS reference population, 20% of the German group and 11% of the Spanish group were overweight or obese, with a tendency toward more overweight or obese children in Germany [25]. The KIGGS study showed that 15% of German children and adolescents aged 3–17 years were overweight and 6% suffered from obesity [17]. Regardless of country, girls had a significantly higher BMI and hip circumference compared to boys. Compared to the reference population, the BMI-SDS and hip circumference SDS of the girls were even above zero [25]. Additionally, the Spanish girls were less active than the Spanish boys, and the same results were found in the overweight or obese Spanish pupils. In contrast, the German children did not show these differences.

PA level has shown to have an influence in this study independently of country and is known to be beneficial for various health outcomes. In addition, it is most effective in adolescents to reduce cardio-metabolic risk as well as overweight and obesity [5,15]. Furthermore, being physically active is related to better quality of life in children and adolescents [49]. Moradell et al. concluded the findings from the international multicenter cross-sectional HELENA study, proving that those adolescents who met the PA guidelines and screentime recommendations had higher intakes of healthy foods (e.g., fruits, vegetables, and dairy products) [3]. Similarly, Lopez-Gil et al. found high associations of PA level and MD scores [36]. PA level has been associated with food choice, and cereals, fruits, and vegetables appeared more likely in the diet of active adults and children [50]. Lazarou et al. (2010) found a healthy diet in children who maintained high levels of PA, which was also found in [36,51,52]. Ottevaere et al. found higher fruit intake in most active males compared to the lowest PA group [53]. The German KIGGS study (2nd wave) found associations between PA levels and consumption of healthy food and beverages in German children and adolescents [52].

In addition to lower PA level in the Spanish girls, the results of this study demonstrate a significant difference in PA during the week and weekend with higher PA at weekdays. This suggests that school hours may be an ideal setting for increasing PA levels and decreasing sedentary activity, since children spend most of their time in this environment [54]. Moreover, it could be used to advise them on how to be physically active during the weekend. The school rhythm may have influenced PA behavior [55]. In Spain, school days generally end at 5 pm compared to German school days which generally end at 3 pm. This difference in time might explain the PA results in this study. These results reflect the huge potential in using long school days for intermitted PA, since sedentary time is increased the longer children are at school during the week [56]. Furthermore, the school setting may be an ideal setting for increasing PA levels. There might be a possibility of developing outdoor physical education programs. For example, these programs could focus on food sustainability from an early age to contribute to expanding responsible food consumption habits while promoting physical activity in the natural environment.

## 5. Limitations

Since both studies have been developed independently, recruitment and measurement protocols differed. With regard to nutrition, a full comparison of the KIDMED was not possible, since three questions were skipped, for example, more than half of the German children did not know which oil was being used at home, as sunflower oil and butter are also very common for cooking in Germany. Furthermore, due to the focus and time frame of the German study, no validation of the German translation of the questionnaires was performed. A direct comparison of physical activity between both countries was not possible since accelerometers worn at different body parts (hip vs. wrist) and different types of accelerometers were used. Therefore, the PA values for each country were calculated separately by tertiles.

## 6. Conclusions

In summary, Spanish children had higher adherence and quality regarding MD compared to German children. Huge differences were detected in typical MD habits, such as fruit-, vegetables-, fish-intakes, and dairy products. In contrast, German children had higher intakes of fast food, processed bakery goods, candies, and sweet beverages. Independently of country, for girls, children with lower BMI-SDS and children with higher PA level were related with better diet quality. However, since girls of both countries had higher BMI-SDS and the Spanish girls were less active than boys, they are at risk of poorer diet quality. Knowing that the majority of the pupils in both countries spent most of their daytime in schools including their lunch, a possible way to influence and change habits on PA and nutrition seems apparent. 

## Figures and Tables

**Table 1:** Country characteristics divided by gender.

	Germany			Spain			Gender		Country		Gender × Country	
	Total	Boys	Girls	Total	Boys	Girls	*p*	η^2^	*p*	η^2^	*p*	η^2^
*N*	152	82	70	182	86	96						
Age (years)	11.4 ± 0.6	11.5 ± 0.6	11.3 ± 0.6	11.4 ± 0.6	11.4 ± 0.7	11.5 ± 0.6	0.583	<0.01	0.908	<0.01	0.025	0.02
Height (m)	1.50 ± 0.08	1.50 ± 0.08	1.50 ± 0.08	1.49 ± 0.08	1.48 ± 0.08	1.50 ± 0.08	0.319	<0.01	0.076	0.01	0.335	<0.01
Weight (kg)	45.6 ± 12.5	44.3 ± 11.6	47.0 ± 13.4	43.9 ± 11.7	42.2 ± 11.4	45.4 ± 11.7	0.027	0.01	0.157	0.01	0.855	<0.01
BMI (kg/m^2^)	20.0 ± 4.3	19.5 ± 4.0	20.5 ± 4.5	19.6 ± 3.9	19.1 ± 3.8	20.1 ± 3.9	0.022	0.02	0.363	<0.01	0.941	<0.01
Hip circumference (cm)	82.1 ± 10.2	80.3 ± 9.5	84.2 ± 10.7	82.1 ± 9.2	79.8 ± 8.9	84.2 ± 9.0	<0.001	0.05	0.778	<0.01	0.844	<0.01

**Table 2:** PA level distribution for gender and BMI group by country.

	Germany				Spain			
	PA Level				PA Level			
	Low	Medium	High	*p*	Low	Medium	High	*p*
Boys (*n)*	25	18	16	0.091	18	28	40	<0.001
	42.4%	30.5%	27.1%		20.9%	32.6%	46.5%	
Girls (*n*)	15	22	25		43	32	21	
	24.2%	35.5%	40.3%		44.8%	33.3%	21.9%	
Normal weight (*n*)	32	33	31	0.740	49	54	59	0.014
	33.3%	34.4%	32.3%		30.2%	33.3%	36.4%	
Overweight (*n*)	8	7	10		12	6	2	
	32.0%	28.0%	40.0%		60.0%	30.0%	10.0%	

PA: Physical activity.

**Table 3:** KIDMED questions by country.

	Germany (%)	Spain (%)	*p*
Fruit or fruit juice daily	70.2	91.2	<0.001
Second serving of fruit daily	57.0	67.0	0.062
Fresh or cooked vegetables daily	56.7	75.3	<0.001
Fresh or cooked vegetables > 1/day	31.3	33.0	0.751
Regular fish consumption (at least 2–3/week)	34.9	81.3	<0.001
>1/week fast-food (hamburger) restaurant	53.0	12.1	<0.001
Pulses > 1/week	71.5	81.3	0.035
Cereal or cereal product for breakfast	92.0	81.9	0.007
No breakfast	45.7	18.1	<0.001
Dairy product for breakfast	80.7	95.6	<0.001
Commercially baked goods or pastries for breakfast	44.7	27.5	0.001
Two yoghurts and/or 40 g cheese daily	49.3	69.2	<0.001
Sweets and candy several times a day	52.7	3.3	<0.001

**Table 4:** IASE and its variables by country.

	Germany		Spain			
	Mean ± SD	*n*	Mean ± SD	*n*	*p*	Hedges’ g
IASE	4 6.0 ± 10.0	144	78.3 ± 7.9	180	<0.001	−3.62
Cereal or cereal products per day	7.33 ± 2.43	149	9.55 ± 1.79	182	<0.001	−1.05
Fresh or cooked vegetables per day	4.17 ± 1.82	150	9.00 ± 1.87	182	<0.001	−2.60
Fruit or fruit juice per day	4.65 ± 1.81	150	9.67 ± 1.13	182	<0.001	−3.40
Dairy products per day	5.03 ± 2.51	150	9.92 ± 0.52	182	<0.001	−2.82
Meat per week	7.26 ± 2.74	146	7.73 ± 2.39	182	0.185	−0.18
Pulses per week	5.61 ± 3.97	148	8.41 ± 2.24	181	<0.001	−0.89
Cold-processed meats per week	3.30 ± 3.08	147	4.86 ± 2.56	181	<0.001	−0.56
Sweets and candy per week	3.12 ± 2.65	149	5.07 ± 2.19	182	<0.001	−0.81
Sugary beverages per week	3.74 ± 3.15	149	6.16 ± 2.73	181	<0.001	−0.82
Diet-variety	1.55 ± 1.44	152	7.96 ± 1.60	182	<0.001	−4.19

IASE: Healthy feeding indicator.

## Data Availability

The datasets used and/or analyzed during the current study are available from the corresponding author on reasonable request.

## Study Registration

Germany: German clinical trials: REBIRTH active school (DRKS-ID der Studie: DRKS00012371).
